# Genomic characterization of intrinsic and acquired resistance to cetuximab in colorectal cancer patients

**DOI:** 10.1038/s41598-019-51981-5

**Published:** 2019-10-25

**Authors:** Steven M. Bray, Jeeyun Lee, Seung Tae Kim, Joon Young Hur, Philip J. Ebert, John N. Calley, Isabella H. Wulur, Thejaswini Gopalappa, Swee Seong Wong, Hui-Rong Qian, Jason C. Ting, Jiangang Liu, Melinda D. Willard, Ruslan D. Novosiadly, Young Suk Park, Joon Oh Park, Ho Yeong Lim, Won Ki Kang, Amit Aggarwal, Hee Cheol Kim, Christoph Reinhard

**Affiliations:** 1 Eli Lilly and Company, Lilly Research Laboratories, Oncology Discovery Research, Indianapolis, IN USA; 2 Division of Hematology-Oncology, Samsung Medical Center, Sungkyunkwan University School of Medicine, Seoul, Korea; 3 Department of Surgery, Samsung Medical Center, Sungkyunkwan University School of Medicine, Seoul, Korea

**Keywords:** Predictive markers, Cancer, Cancer genomics

## Abstract

Anti-EGFR antibodies are effective in therapies for late-stage colorectal cancer (CRC); however, many tumours are unresponsive or develop resistance. We performed genomic analysis of intrinsic and acquired resistance to anti-EGFR therapy in prospectively collected tumour samples from 25 CRC patients receiving cetuximab (an EGFR inhibitor). Of 25 CRC patients, 13 displayed intrinsic resistance to cetuximab; 12 were intrinsically sensitive. We obtained six re-biopsy samples at acquired resistance from the intrinsically sensitive patients. *NCOA4–RET* and *LMNA–NTRK1* fusions and *NRG1* and *GNAS* amplifications were found in intrinsic-resistant patients. In cetuximab-sensitive patients, we found KRAS K117N and A146T mutations in addition to BRAF V600E, AKT1 E17K, PIK3CA E542K, and *FGFR1* or *ERBB2* amplifications. The comparison between baseline and acquired-resistant tumours revealed an extreme shift in variant allele frequency of somatic variants, suggesting that cetuximab exposure dramatically selected for rare resistant subclones that were initially undetectable. There was also an increase in epithelial-to-mesenchymal transition at acquired resistance, with a reduction in the immune infiltrate. Furthermore, characterization of an acquired-resistant, patient-derived cell line showed that PI3K/mTOR inhibition could rescue cetuximab resistance. Thus, we uncovered novel genomic alterations that elucidate the mechanisms of sensitivity and resistance to anti-EGFR therapy in metastatic CRC patients.

## Introduction

Cetuximab-based chemotherapy has demonstrated survival benefit in patients with metastatic colorectal cancers (CRCs) over the past decade^1–4^. Cetuximab binds to the extracellular domain of the epidermal growth factor receptor (EGFR), which inhibits the RAS–RAF–mitogen-activated protein kinase 1 (MAPK1) and the v-akt murine thymoma viral oncogene homolog 1 (AKT1) axis, the pathways involved in cell proliferation, cell survival and tumour invasion^5^.

In approximately 40% of CRC patients, tumours harbour mutations in the v-Ki-ras2 Kirsten rat sarcoma viral oncogene homolog (*KRAS*), mainly in codons 12, 13, and 61^6,7^. *KRAS* mutations are the key negative predictive factors for cetuximab-based treatment in mCRC patients^8,9^. Although patients with *KRAS* wild-type (wt) CRC tumours are known to be responsive to cetuximab-based treatment, up to 65% of patients with *KRAS* wt tumours are resistant to anti-EGFR monoclonal antibodies^10^. Aberrations in other effectors of the EGFR signalling cascade (*PIK3CA*, *PTEN*, and *NRAS*) have been suggested to affect the primary response to cetuximab sensitivity^11–13^. In addition, *MET* amplification has been detected in CRC patients who initially responded to cetuximab but eventually acquired resistance^14^; however, *MET* amplification occurs only in 1% of CRC patients. Limited progress has been made in understanding the mechanism of resistance to cetuximab, particularly in patients who initially respond to cetuximab but acquire resistance during cetuximab-based chemotherapy.

In this study, we aimed to evaluate intrinsic and acquired resistance to anti-EGFR therapy in prospectively collected tumour samples of *KRAS* wt metastatic CRC (mCRC) patients who were administered cetuximab-containing regimens in real-world clinical care. We also attempted to obtain tumour tissues at tumour progression to investigate the genomic aberrations responsible for acquired resistance. Finally, we established patient-derived tumour cells from the tissues at the time of acquired resistance to cetuximab to explore alternative treatment regimens for these patients.

## Results

### Patient cohort

**Figure 1:**
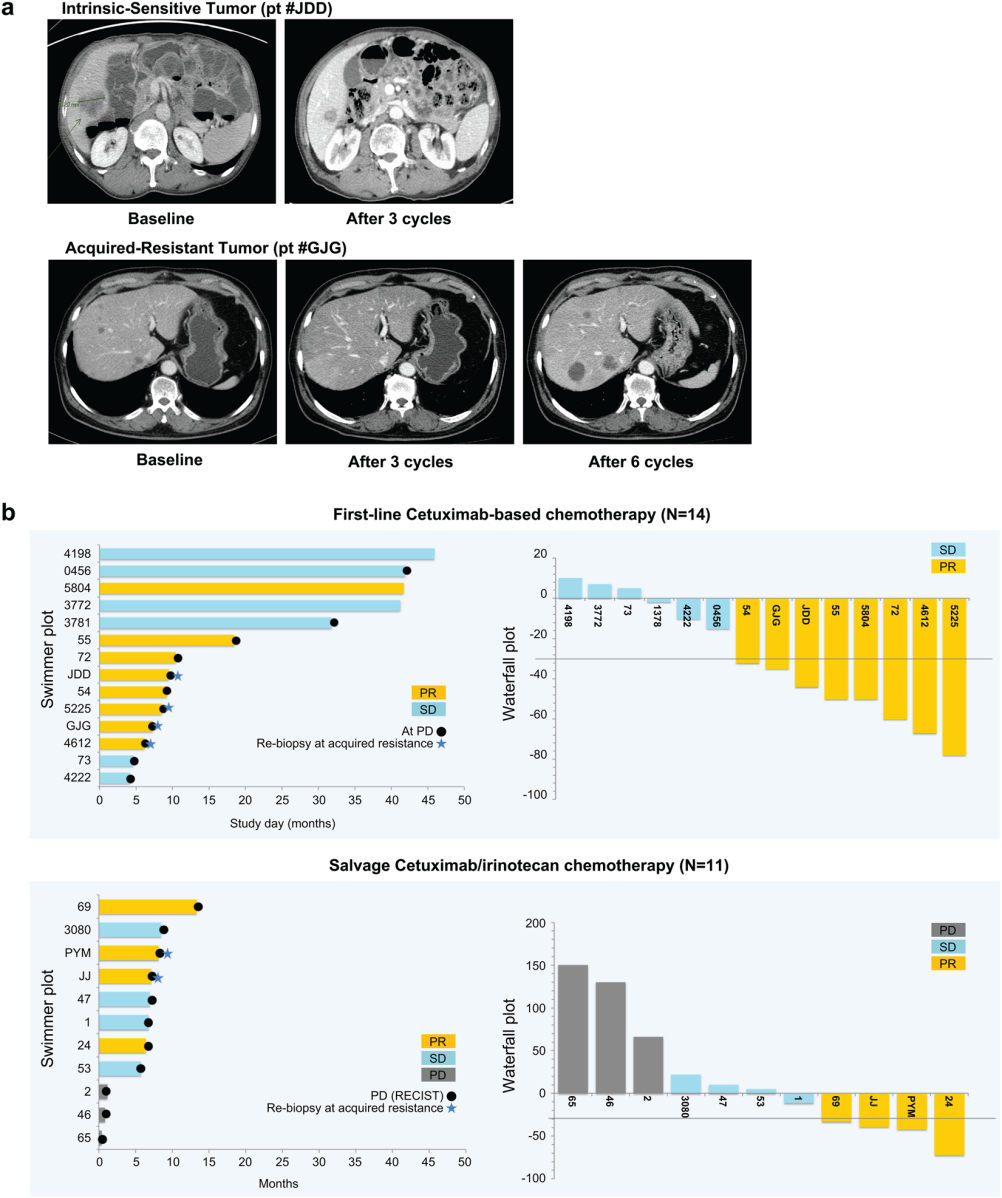
Clinical response to cetuximab. (**a**) Representative computed tomography scans from cetuximab intrinsic-sensitive and acquired-resistant colorectal cancer (CRC) patients. (**b**) Horizontal bar plots represent time (months) for which patients were on cetuximab treatment until progressive disease (PD) (black dots) for first-line cetuximab-based chemotherapy (n = 14) (top) or salvage cetuximab/irinotecan chemotherapy (n = 11) (bottom). Orange, blue, and grey bars indicate partial response (PR), stable disease (SD), and PD, respectively. Blue stars indicate successful re-biopsy in patients who achieved PR and then developed acquired resistance. Vertical waterfall bar plots in the right panels show the percent change in tumour size (y-axis) from baseline during cetuximab treatment. As defined by RECIST criteria, patients who achieved a shrinkage in tumour size of >30% were classified as PR and ‘intrinsic sensitive’ for our study.

Genomic profiling was performed on 25 metastatic CRC patients treated with cetuximab-based chemotherapy (Table 1). All baseline tumours used for sequencing were from the primary tumour site. DNA and RNA were extracted for whole-exome and transcriptome sequencing, as well as copy number (CN) analysis using genotype arrays (Table S1). Fourteen patients were administered first-line cetuximab/FOLFIRI or cetuximab/FOLFOX for metastatic disease, and 11 patients were administered cetuximab/irinotecan as a salvage treatment. Patients who showed stable disease (SD) or progressive disease (PD) following cetuximab treatment were categorized as ‘intrinsic-resistant’, and patients with complete response (CR) or partial response (PR) were categorized as ‘intrinsic-sensitive’ (Fig. 1a). Moreover, among patients showing intrinsic sensitivity to cetuximab, those who developed resistance to cetuximab during cetuximab-based treatment or within 2 months following the completion of cetuximab-based treatment were defined as ‘acquired-resistant’. In the first-line setting (n = 14), eight (57.1%) patients achieved a confirmed PR (Fig. 1b). Of the 11 patients administered irinotecan/cetuximab as a salvage treatment, four (36.4%) achieved a PR (Fig. 1b). Of these 12 cetuximab-sensitive patients (eight in the first-line setting and four in the salvage setting), we successfully obtained re-biopsies at the time of acquired resistance in six patients (blue stars; four in the first-line setting and two in the salvage setting; Fig. 1b). The re-biopsy sites at acquired resistance following the initial response to cetuximab were as follows: colon, n = 2; peritoneal seeding, n = 2; bone, n = 1; and liver, n = 1.

**Table 1:** Clinicopathological characteristics of all enrolled patients (n = 25).

Variables		n	%
Sex	Male	17	68.0
	Female	8	32.0
Age (range)		63	25–85
ECOG performance status	1	25	100
Pathological differentiation	W/D adenocarcinoma	1	4.0
	M/D adenocarcinoma	17	68.0
	P/D adenocarcinoma	2	8.0
	Mucinous adenocarcinoma	4	16.0
	Signet ring cell carcinoma	1	1.0
Regimens including cetuximab	Irinotecan + cetuximab (+3^rd^ line)	11	44.0
	FOLFIRI + cetuximab (1^st^ line)	10	40.0
	FOLFOX + cetuximab (1^st^ line)	4	16.0
*KRAS* mutational status (direct sequencing)	Wild-type	25	100

*W/D: well differentiated; M/D: moderately differentiated; P/D: poorly differentiated.

### Intrinsic-resistant tumours

**Figure 2:**
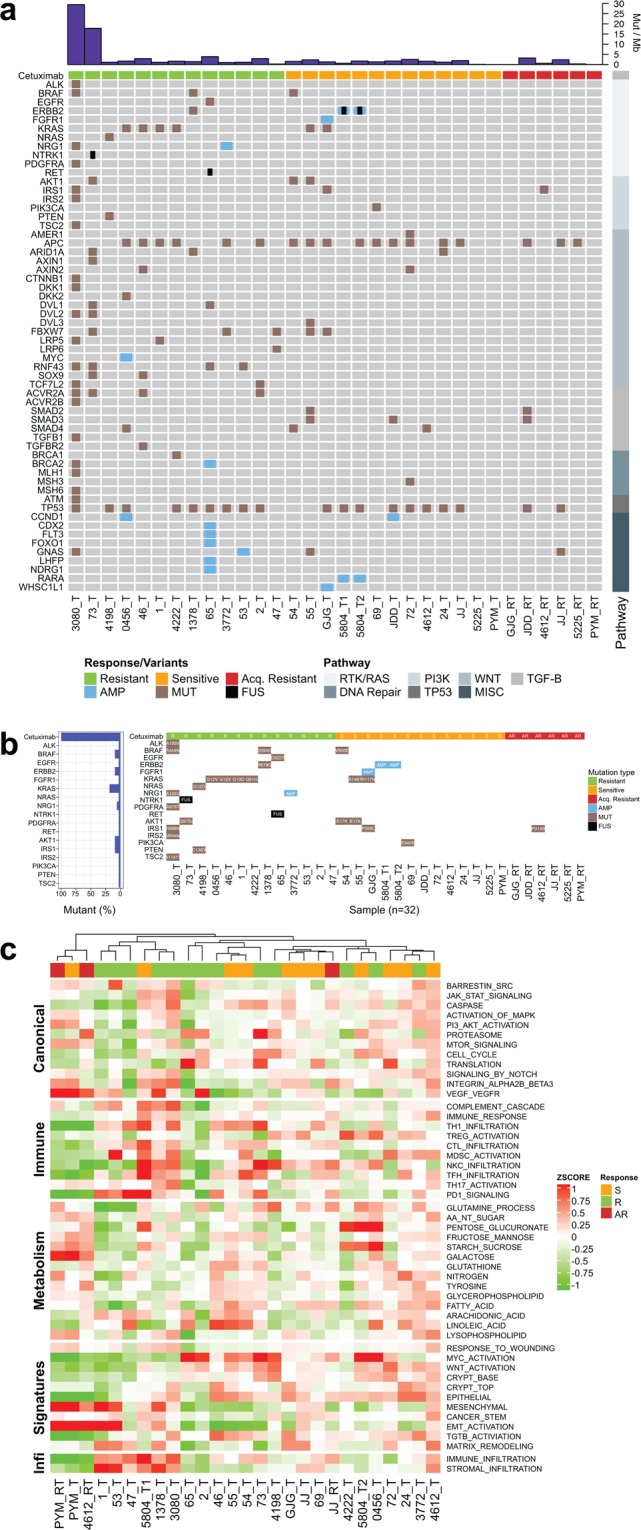
Landscape of somatic variants and gene set expression signatures in sensitive, resistant, and acquired-resistant tumours. (**a,b**) Somatic variants identified in genes grouped by molecular pathways. MUT, non-synonymous single nucleotide or small indel mutation; FUS, gene fusion; AMP, copy number amplification (CN ≥4). The top bar chart shows the tumour mutation burden of non-synonymous somatic variants. Amino acid change is shown for a subset of genes in (**b**). (**c**) Hierarchical clustering of gene set expression signatures for a panel of published molecular signatures (see Methods). The Z score represents both the magnitude and relative direction of a signature’s expression. Baseline tumour (T), acquired-resistant tumour (RT).

The genomic landscape of the intrinsic-sensitive, intrinsic-resistant, and acquired-resistant tumours using exome, transcriptome, and CN analysis is shown in Fig. 2. Genetic alterations (Fig. 2a,b) in RAS/RAF pathway regulators were frequently observed in intrinsic-resistant tumours. Although all patients were selected based on direct sequencing of *KRAS* at the time of cetuximab administration, whole-exome sequencing identified four additional patients with KRAS mutations (two with G12V and once each with G13D and Q61H). An NRAS G12D mutant tumour also showed cetuximab resistance. Nearly all tumours profiled showed a low somatic mutation burden (0.0–3.9 non-silent Mut/Mb), which is consistent with our previous classification of all patient tumours as microsatellite stable. Notably, the two exceptions with high mutation burden (patient #3080, 29.5 Mut/Mb; patient #73, 17.8 Mut/Mb) showed cetuximab resistance (Fig. 2a, top). Consistent with the results of recent studies, we also detected point mutations in *ERBB2* and *PDGFRA* that likely confer cetuximab resistance^15,16^. The PDGFRA A978T variant is near the PDGFRA kinase domain and is close to the R981H mutation, which has been shown to confer cetuximab resistance in an earlier study^15^. In that same study, a xenograft model carrying the PDGFRA R981H mutation treated with imatinib in combination with cetuximab showed increased efficacy, suggesting that PDGFRA kinase inhibitors might be useful for treating cetuximab-resistant CRC. One somatic EGFR mutation, D522Y, was also identified in the EGFR extracellular domain of an intrinsic-resistant tumour; however, residue 522 is not in the EGFR cetuximab-binding domain. Its impact on cetuximab affinity^17^ requires further validation.

**Figure 3:**
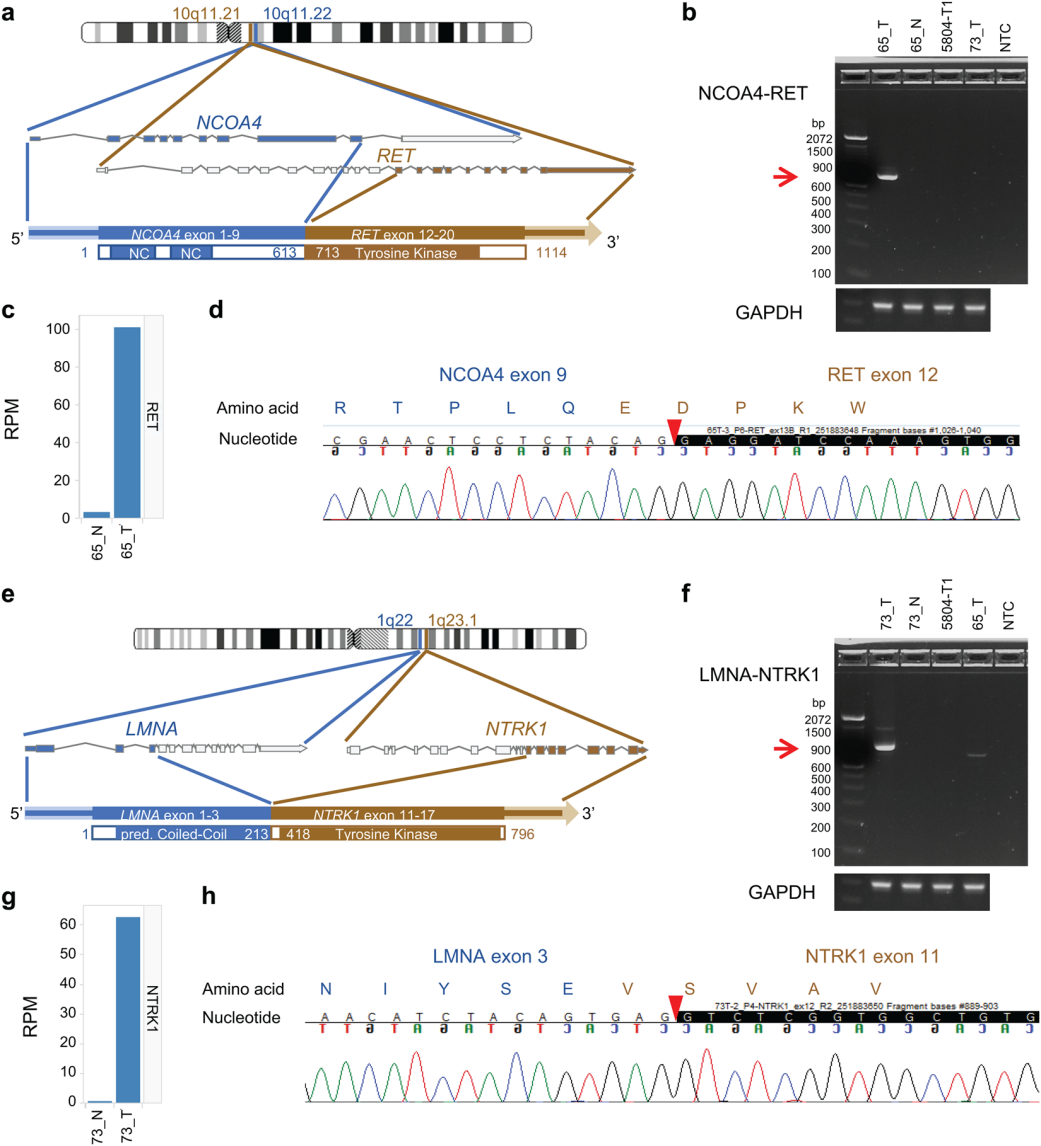
Characterization of *NCOA4–RET* and *LMNA–NTRK1* gene fusions. (**a**,**e**) Schematic depiction of the gene fusion detected by RNA-Seq, indicating the genomic position, resulting fusion mRNA, and predicted protein consequences (with key protein domains indicated). (**b**,**f**) RT-PCR of RNA from the tumour or adjacent normal tissue with primers upstream and downstream of the gene fusion breakpoint. NTC, no template control. Red arrows indicate the size of the expected PCR product. (**c**,**g**) Overexpression of the 3′ fusion gene partner in the tumour relative to the adjacent normal tissue. RPM, reads per million. (**d**,**h**) Sanger sequencing of the RT-PCR product from (**b**) and (**f**). The red arrowheads indicate the position of the fusion junction and in-frame amino acid sequence.

Using RNA sequencing data, we also identified two in-frame gene fusions (Tables S2, S3, and Fig. 3), including *NCOA4–RET* and *LMNA–NTRK1* fusions. These fusions were both confirmed to be tumour-specific (Fig. 3b,f) and preserved the tyrosine kinase domain in the 3′ fusion partners RET and NTRK1, respectively (Fig. 3a,d,e,h). Expression analysis further revealed the dramatic upregulation of *RET* and *NTRK1* fusion transcripts in the tumours relative to their matched normal tissue (Fig. 3c,g). While *NTRK1* fusions have been reported in cetuximab-resistant patients^18,19^, this is the first clinical report demonstrating *RET* fusion as the putative cause of cetuximab resistance. RTK fusions, including *RET* and *NTRK1* fusions, have been identified in ~0.4% of CRC patients^16^.

Genome-wide CN analysis of the 13 intrinsic-resistant tumours revealed *NRG1* and *GNAS* amplifications in CRC tumours without any aberrations in RAS/RAF pathway genes (Fig. 2a). Neuregulin 1 (*NRG1*; also known as heregulin), encodes an EGF-like signalling molecule that binds and activates ERBB2/3 heterodimers. The high CN of *NRG1* as a resistance marker is consistent with previous findings of higher NRG1 protein and RNA expression levels corresponding to cetuximab resistance^13,20^. *GNAS* encodes the Gs-alpha subunit of G-proteins, and *GNAS* amplification or point mutations have been identified in approximately 10% of CRC tumours, often coinciding with *KRAS* mutations^21^. *GNAS* gain-of-function variants result in elevated cAMP and ERK/MAPK activation^22^. GNAS was also predicted to play a role in resistance to BRAF + EGFR inhibition in *BRAF* V600E mutant CRC^23^. *GNAS* amplification in patient #53 correlates with >6-fold increased RNA expression.

Furthermore, to explore whether changes in the expression of known molecular pathways contribute to cetuximab resistance, we performed gene set enrichment analysis (Figs 2c, S1). We identified no consistently up- or downregulated pathways for differentiating sensitive from resistant tumours in well-characterized pathways in CRC. Overall, our genomic analyses identified potential resistance-causing alterations in 11 of the 13 intrinsic-resistant tumours. These variants appear to bypass the anti-EGFR blockade by activating alterations in RAS/RAF pathway genes.

### Intrinsic-sensitive tumours

For cetuximab-sensitive tumours, there were no major aberrations in the RAS pathway in any of the 12 tumours (Fig. 2a,b). Two patients showed KRAS K117N and A146T mutations, respectively, which are in *KRAS* exon 4 rather than in the more dominant exon 2 (G12/13)^24,25^. We also found other RTK/RAF and PI3K/AKT pathway mutations in the cetuximab-sensitive cohort, including BRAF V600E (n = 1), AKT1 E17K (n = 2), PIK3CA E542K (n = 1), *FGFR1* amplification (n = 1), and *ERBB2* amplification (n = 2). In patient #5804, in addition to an *ERBB2* amplification in primary and metastatic lesions, we also discovered an *ERBB2–MED24* fusion present only in liver metastasis (Table S2 and Fig. S2). The fusion lacks the ERBB2 kinase domain, and expression analysis indicates that it did not contribute to *ERBB2* expression (data not shown), leading us to conclude that it might not be a driver mutation. Extended analysis of *ERBB2* fusion transcripts in TCGA RNA-Seq data shows a close association of *ERBB2* fusions with high *ERBB2* CN, possibly a result of secondary passenger events in the amplification process (Table S4).

### Acquired-resistant tumours

Among the 12 cetuximab-sensitive patients, 11 developed acquired resistance following the response to cetuximab, and we obtained follow-up resistant tumour specimens from six patients (Fig. 1b). We successfully profiled all six acquired-resistant tumours using exome sequencing, but due to low tissue volume and quality, we were only able to analyse five for CN variants and three with RNA-Seq (Table S1). We compared the variant and CN profiles between the pre-treated baseline and re-biopsied tumours as shown in Fig. 2. Previously described cetuximab resistance-related genomic aberrations were not found in the acquired-resistant tumours, and novel variants did not immediately suggest any apparent resistance mechanisms.

**Figure 4:**
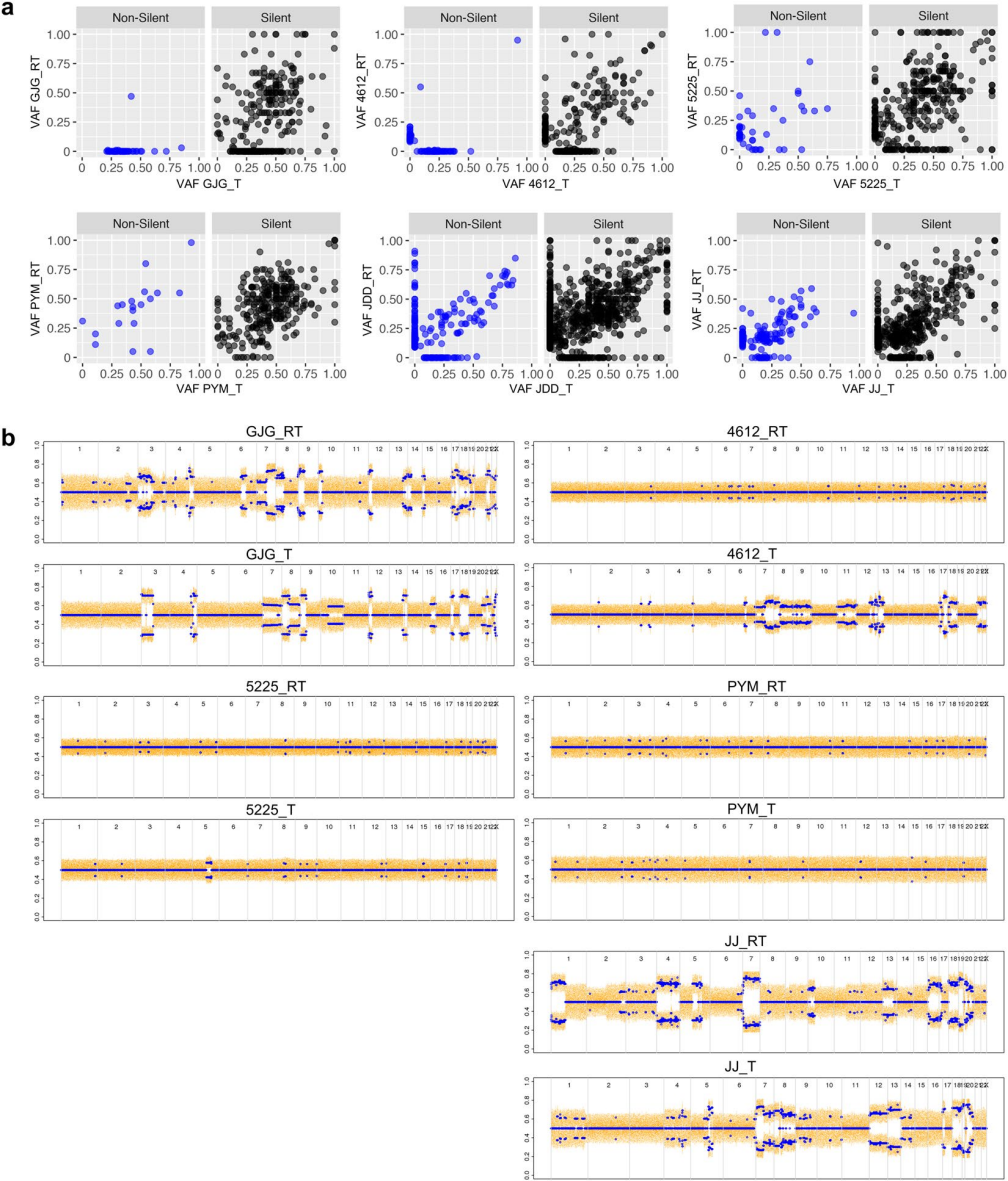
Variant and copy number changes in sensitive and acquired-resistant tumour pairs. (**a**) Variant allele frequency (VAF) of somatic mutations detected in baseline (T) versus acquired-resistant (RT) tumours. Variants were grouped as non-silent (blue) and silent (black) based on the predicted impact on protein sequence. (**b**) B-allele frequency (BAF) plots show regions of genomic imbalance from copy number variants for each chromosome (1–22) of T and RT.

We plotted and compared the variant allele fraction (VAF) of silent and non-silent variants in six pre- and post-treatment paired samples (Fig. 4a). The comparison revealed both an extreme loss (variants found along the x-axis, VAF = 0 for acquired-resistant tumour) and simultaneous gain (variants found along the y-axis, VAF = 0 for baseline tumour) of somatic variants in acquired-resistant tumours at the time of disease progression. Of note, patient #GJG showed no gain of non-silent acquired-resistant variants. These results suggest that cetuximab exposure dramatically selected for rare resistant subclones of the baseline tumour that were undetectable at the start of the treatment. These observations were further validated by CN array B-allele frequency (BAF) analysis in the re-biopsied resistant samples (Fig. 4b). The BAF plots showed many loci with BAF imbalance in the baseline-sensitive tumours, which were ‘lost’ in the acquired-resistant tumours. For example, chromosome 10 in patient #GJG showed BAF skewing in the sensitive tumour, which was no longer present in the acquired-resistant tumour. Because tumour evolution is unlikely to restore BAF imbalances as the tumour progresses, we concluded that the acquired-resistant tumour was likely derived from a minor subclone that originally had normal BAF balance at the chromosome 10 locus prior to cetuximab treatment. Patient #4612 showed the most dramatic changes in BAF, which is also consistent with the shift observed in its non-silent VAF.

**Figure 5:**
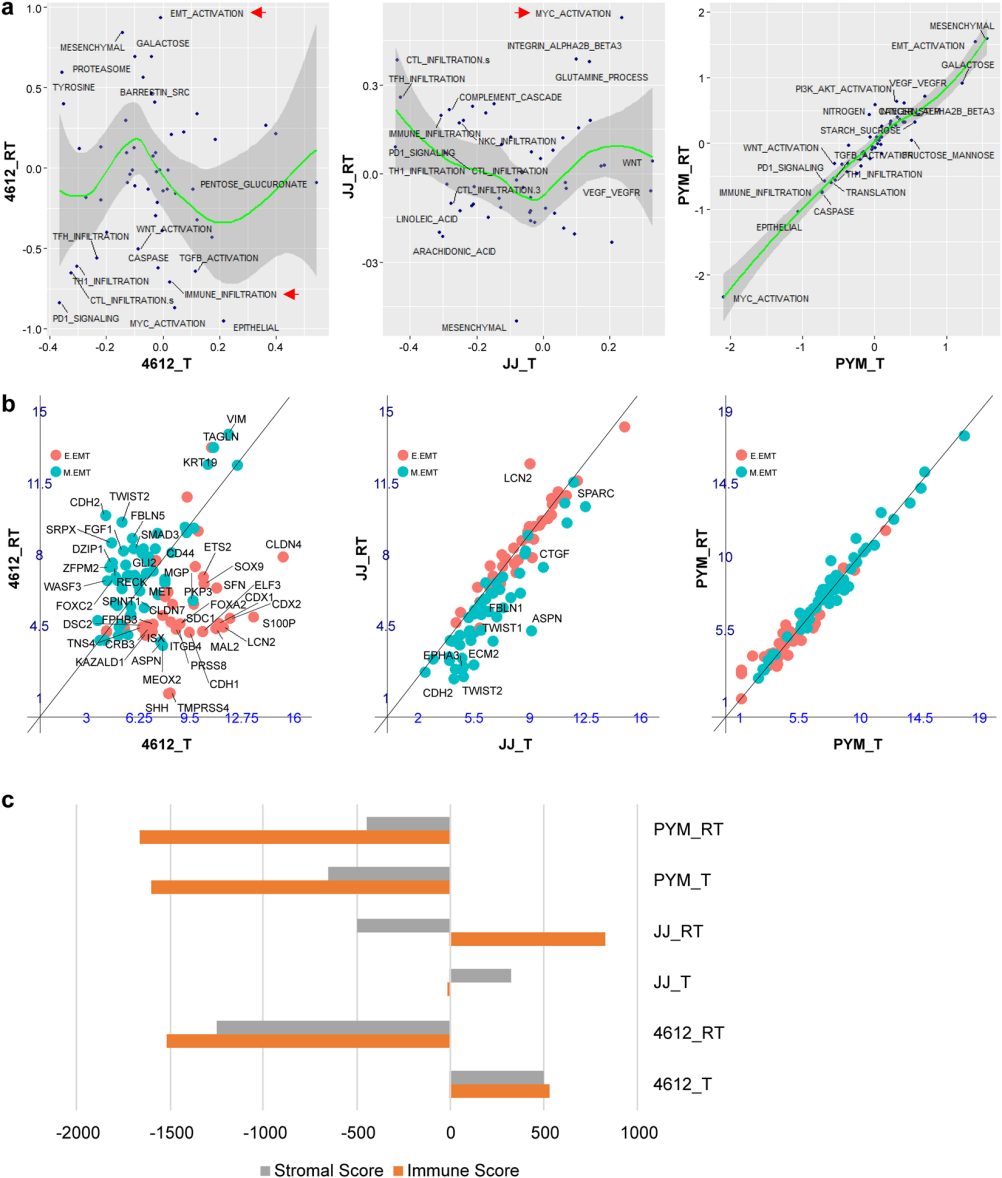
Gene expression signatures in sensitive and acquired-resistant tumour pairs. Analysis of RNA expression signatures and enrichment scores in sensitive versus acquired-resistant tumour pairs. Baseline tumour (T), acquired-resistant tumour (RT). (**a**) Pathway enrichment Z-scores plotted for each molecular pathway expression signature. The green line represents a smooth local regression LOESS curve, and grey shading highlights the 95% confidence interval around the curve. Pathways with the highest enrichment Z-scores in each panel are labelled. Red arrows highlight pathways of interest. (**b**) Expression (log2) of individual genes that comprise the epithelial (E.EMT) and mesenchymal (M.EMT) gene expression signature. A diagonal line is plotted for comparison. The EMT signature genes showing >4-fold change difference of expression between baseline tumour and acquired-resistant tumour are labelled. (**c**) Gene expression signatures for stromal and immune cell tumour infiltration were quantified using the ESTIMATE tool (see Methods). Orange bars represent scores for immune cell infiltration and grey bars, scores for stromal cell infiltration.

To evaluate whether clonal selection of acquired-resistant tumours would be reflected in the activation of specific CRC-related transcription pathways, we looked for enrichment scores for gene expression pathways between baseline and acquired-resistant tumour pairs (pts. #JJ, #4612, #PYM) (Fig. 5a). Patient #4612 showed enrichment for several molecular pathways, including an increase in epithelial-to-mesenchymal transition (EMT), with a reduction in the immune infiltrate. To explore these findings further, we compared the expression of all genes constituting the EMT signature and found a clear upregulation of mesenchymal markers compared to epithelial markers in the acquired-resistant tumour from patient #4612 but not patient #JJ or patient #PYM (Fig. 5b). Using the ESTIMATE tool to quantify the immune and stromal infiltration, we also confirmed that patient #4612 had a dramatic reduction in immune and stromal scores (Fig. 5c)^26^. Additional signature analyses of EMT and immune and metabolic pathways in patient #4612 are depicted in Supplementary Fig. S3. Patient #JJ also demonstrated a drop in the stromal infiltration score but instead showed an increase in the immune infiltration score at resistance (Fig. 5c). In addition, patient #JJ also showed an increase in MYC signalling in his parental versus acquired-resistant samples (Fig. 5a). No clear change was observed in the expression signatures for patient #PYM. These results suggest that multiple mechanisms are involved in clonal evolution under selective pressure by cetuximab.

### *In vitro* analysis of an acquired-resistant, patient-derived cell line

**Figure 6:**
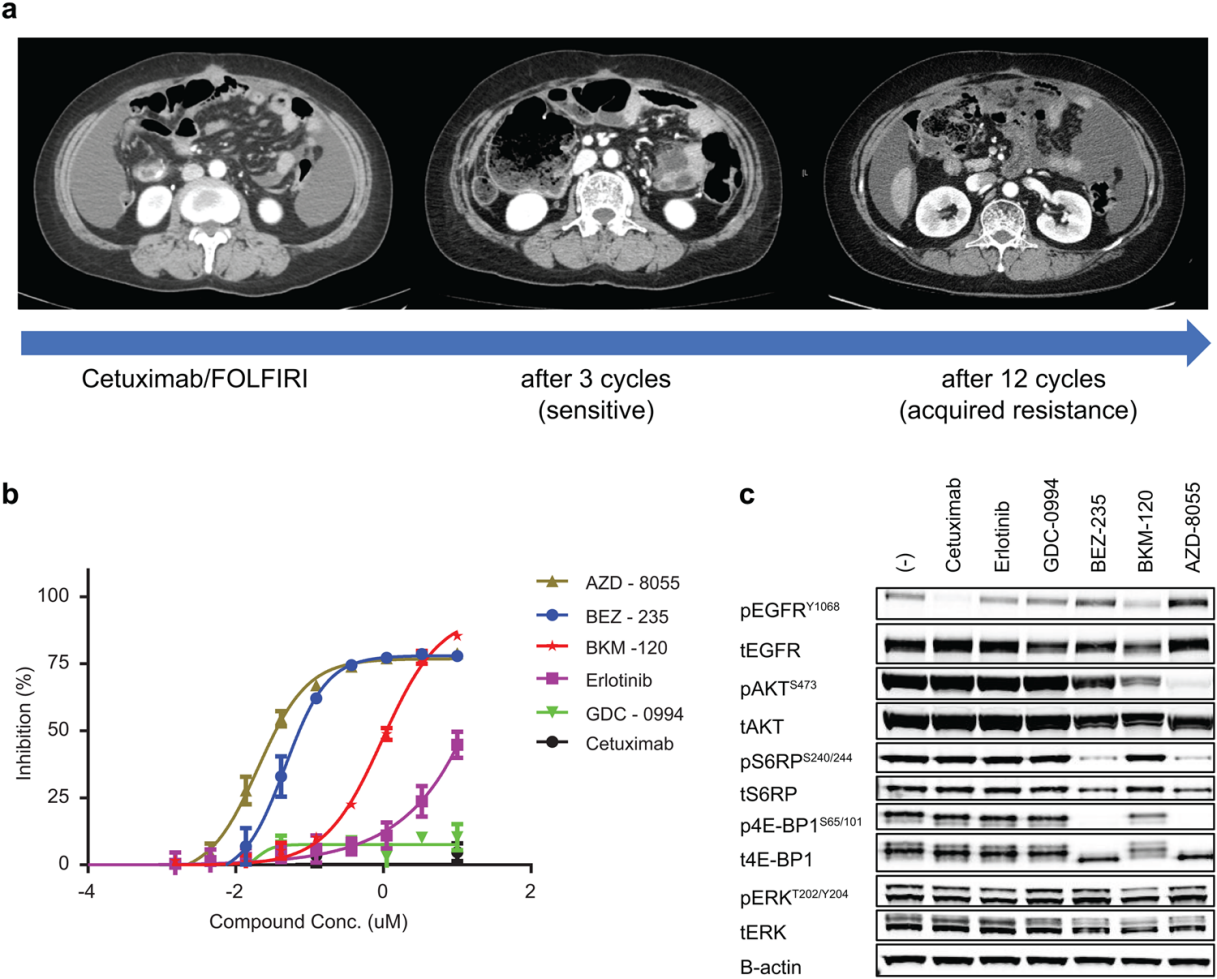
Sensitivity of a patient-derived cell line from patient #4612 to PI3K/mTOR inhibition. (**a**) Computed tomography scans for patient #4612 at baseline and following 3 and 12 cycles of cetuximab treatment. A patient-derived cell (PDC) line was cultured from the acquired-resistant tumour (malignant ascites, which developed following 12 cycles). (**b**) Dose-dependent inhibition of PDC cell proliferation by 5-day treatments with cetuximab (EGFR), erlotinib (EGFR), GDC-0994 (Erk1/2), BEZ-235 (dual PI3K/mTOR), AZD-8055 (mTOR), and BKM-120 (pan-PI3K). Results from four independent experiments are shown. The IC_50_ values of AZD-8055, BEZ-235, and BKM-120 are 0.02, 0.047, and 0.971 µM, respectively. (**c**) Western blot expression of basal and 24 h treatments of PDC. Cetuximab concentration, 75 µg/mL; other compounds, 1 µM. The experiments were repeated thrice.

To investigate the development of a treatment strategy for overcoming the acquired resistance to EGFR-targeted therapy, we established a cetuximab-resistant, patient-derived cell (PDC) line from peritoneal seeding tissue of patient #4612 at acquired resistance. This 60-year old female patient initially had *KRAS* wt, *NRAS* wt, and *TP53* mt colorectal mucinous adenocarcinoma with peritoneal seeding and showed a dramatic response to cetuximab for 5 months; however, during cetuximab treatment, she was found to have peritoneal seeding (Fig. 6a).

To confirm that the PDC line originated from the acquired-resistant tumour, we measured the expression of EMT markers and performed Sanger sequencing of two acquired-resistant mutations. We correspondingly observed a loss of E-cadherin expression and gain of N-cadherin, vimentin, and AXL expression in the acquired-resistant tumour for patient #4612 (Table S5). Similarly, we confirmed the presence of the acquired variants *GTF2H5* and *SLC17A1* (Table S6).

Cell proliferation assays using the PDC showed resistance to EGFR inhibition with cetuximab or erlotinib as expected (Fig. 6b). We next tested whether the cetuximab-resistant PDC would be sensitive to the inhibition of the MEK/ERK pathway, which has been suggested as a cetuximab resistance mechanism in previous cell line and animal studies^27,28^. However, the Erk1/2 inhibitor GDC-0994 did not suppress the growth of the PDC line (Fig. 6b). Further analysis of the acquired mutations and RNA expression in the acquired-resistant tumour for patient #4612 revealed both an acquired IRS1 P313S variant (Fig. 2b), as well as a ~5-fold decrease in the expression of the PI3K regulatory subunit *PIK3R1*, leading us to hypothesize that the PI3K/mTOR pathways might be dysregulated in the resistant tumour. Although canonical PI3K/mTOR expression signatures were not considerably altered in our gene signature analysis closer investigation of individual gene expression revealed greater expression shifts relative to baseline for patient #4612 (Fig. S4), which could impact protein-level pathway activation that is not captured by the canonical RNA expression signatures. Indeed, the PDCs were sensitive to dual PI3K/mTOR (BEZ-235), mTOR (AZD-8055), and to a lesser degree, pan-PI3K (BKM-120) treatments. Immunoblot assays for downstream pathway activation also revealed that dual PI3K/mTOR (BEZ-235) and mTOR (AZD-8055) treatments potently inhibited the phosphorylation of S6RP^S240/244^ and 4E-BP1^S65/101^ (Fig. 6c), which was concordant with the inhibition of cell proliferation.

## Discussion

The aim of this study was to evaluate the mechanisms underlying sensitivity and resistance to cetuximab-based chemotherapy in CRC patients. Given the complexity of EGFR crosstalk that affects sensitivity and resistance to anti-EGFR therapy in mCRC as previously described^29^, a simple genomic aberration or pathway may not explain the sensitivity or resistance to cetuximab in mCRC. Comparative genomic analyses suggested that most of the acquired-resistant tumours in our study resulted from a selective outgrowth of a minor clonal variant in the primary tumour that was not detectable at baseline by the standard NGS approach, as suggested earlier^30^. Detailed analysis of intrinsic-resistant tumours revealed several single-nucleotide variants in *KRAS*, *NRAS*, *ERBB2*, and *PDGFRA*, which likely confer resistance. Moreover, we identified *NCOA4–RET* and *LMNA–NTRK1* fusions in two patients who were both refractory to cetuximab-based chemotherapy. The *LMNA–NTRK1* fusion was recently identified as a potential target in cetuximab-resistant tumours^18^. The largest global survey of 17 *RET* fusion-positive mCRC patients demonstrated that all patients with rearranged *RET* had *KRAS* wt, and 10 of them had an *NCOA4–RET* fusion associated with significantly poorer survival compared to the *RET* fusion-negative *KRAS* wt patients^31,32^. This is the first study to detect an *NCOA4–RET* fusion in cetuximab-resistant *KRAS* wt CRC. The possibility that *RET* fusions drive resistance to EGFR inhibition is corroborated by recent studies showing that *RET* fusions confer resistance to osimertinib in non-small cell lung cancer patients^33^. Furthermore, the lung cancer cell line Lc2/ad, which carries a *RET* fusion, shows more resistance to erlotinib compared to SW48 cells, and direct targeting of RET inhibition in Lc2/ad cells can be bypassed by EGFR activation^34,35^.

Novel amplifications of *NRG1* and *GNAS* may predict resistance to cetuximab (both found in *KRAS* wt, *NRAS* wt patients). In the RAS pathway, NRG1 and other EGFR ligands were previously found to play a major role in conferring primary cetuximab resistance in CRC pre-clinical models, although a correlation between NRG1 expression and *NRG1* amplification was not reported^36^. The patient harbouring the *NRG1* amplification (patient #3772) was a 76-year-old woman with poorly differentiated adenocarcinoma, who demonstrated progression after three cycles of cetuximab/irinotecan. NRGs are encoded by four individual genes (*NRG1–4*), and *NRG1* generates six types of proteins (I–VI) in at least 31 isoforms (29). All isoforms contain an extracellular EGF-like domain that induces the activation of ERBB RTKs. Recently, an *NRG1* fusion inducing ERBB3 activation was characterized in lung cancer^37^ and breast cancer^38^; however, *NRG1* amplification has not been characterized in CRC.

In cetuximab-sensitive tumours, we identified several mutations in PI3K/AKT and RTK/RAS pathway genes, which would be expected to confer cetuximab resistance, including variants in *BRAF*, *KRAS*, *PIK3CA*, *AKT1*, *FGFR1*, and *ERBB2*. Our findings suggest that the presence of these specific variants alone is not sufficient to preclude an objective response to cetuximab. A recurrent AKT1 E17K hotspot mutation was found in two out of 25 patients in our cohort (8%). In one study, *AKT1* was found to be mutated in only ~1.5% of TCGA CRCs^39^, suggesting that the mutation frequency is higher in Asian populations. Several of these putative resistance variants co-occurred in three cetuximab-sensitive tumours, indicating that some interplay between the variants may sensitize tumours to cetuximab.

Notably, patient #4612 acquired resistance to cetuximab and demonstrated several interesting genomic features at progression. First, the EMT signature was low at baseline, but was converted to a high EMT subtype at progression to cetuximab, which corresponds with recent findings showing that five of eight paired biopsies exhibited subtype switching to EMT at PD^40^. EMT has been associated with an invasive and metastatic phenotype, as well as drug resistance to both chemotherapy and molecularly targeted agents, while mechanisms to reverse EMT have been shown to re-sensitize some tumours to drug rechallenge^41,42^. Second, the PDC line established at acquired resistance from this patient was sensitive to dual PI3K/mTOR and mTOR inhibition. The re-biopsied tumour at resistance was *PIK3CA* wt but had low *PIK3R1* expression and acquired an IRS1 P313S variant that was not present in the sensitive tumour. The *PIK3R1* gene encodes the p85 regulatory subunit of PI3K, and IRS1 directly binds with p85 following IRS1 tyrosine phosphorylation^43,44^. IRS1 tyrosine phosphorylation is downregulated by the phosphorylation of multiple IRS1 serine residues in an mTOR-dependent manner. Proline-directed serine phosphorylation of human IRS1 Ser312, immediately adjacent to the IRS1 P313S variant, is a key serine residue for IRS1 feedback regulation^43–45^. Thus, low expression of p85 and an inability of IRS1 to respond to negative feedback signals could contribute to higher PI3K/mTOR activity and cetuximab resistance. It is unclear if EMT and acquired-resistant *IRS1*/*PIK3R1* alterations are both contributing to cetuximab resistance in pt. #4612. EMT and *ALK* mutations were recently found to co-exist and separately contribute to resistance in the same crizotinib-resistant tumor lesions. Inhibition of the PI3K/mTOR pathway has also been previously shown to reverse expression of EMT markers, however, reversion of EMT alone does not directly account for blocking cell proliferation as demonstrated in recent studies that have pharmacologically reversed EMT but needed additional drug rechallenge to inhibit tumor growth. Therefore, we propose that sensitivity to PI3K/mTOR inhibitors is most likely due to *IRS1*/*PIK3R1* acquired mutations rather than EMT reversion. Nonetheless, regardless of the specific resistance mechanism, our results clearly show that the patient-derived tumor cells are ‘oncogene addicted’ to PI3K/mTOR activation and therefore inhibition of this pathway is sufficient to overcome cetuximab resistance in this patient.

In summary, we show that *NCOA4–RET* and *LMNA–NTRK1* fusions, along with *NRG1* and *GNAS* amplifications, are potentially novel cetuximab-resistance (intrinsic) alterations in patients with *KRAS* wt CRC. Clonal selection in acquired resistance appears to be common with changes in CRC-related pathways and its microenvironment. We demonstrated that a PDC model derived from an acquired-resistant tumour was successfully suppressed by PI3K/mTOR inhibitors such as AZD-8055 or BEZ-235. The clinical benefit from these molecules should be evaluated in clinical trials, especially in a salvage setting with patients who have acquired resistance to cetuximab.

## Methods

### Patients and samples

Twenty-five mCRC patients administered treatment containing cetuximab were enrolled for the prospective collection of tumour samples. All study participants provided written informed consent. The study was approved by the institutional review board at Samsung Medical Center (IRB# 2013-10-014). The study was conducted in accordance with the Declaration of Helsinki and the Guidelines for Good Clinical Practice. Baseline tumour samples were collected before initiating cetuximab-based chemotherapy in all enrolled patients, and additional tumour samples were obtained at progression. All collected tumour samples were confirmed by our pathologists for tumour content and characteristics. All hematoxylin and eosin-stained slides were also reviewed. Patient age at diagnosis, sex, Eastern Cooperative Oncology Group performance status, number of involved organs, metastatic site, and chemotherapy data were collected.

### Study procedure and statistical analysis

Biopsies were performed before cetuximab-based chemotherapy regimens. DNA and RNA were extracted for whole-exome and transcriptome sequencing, as well as CN analysis using genotype arrays. Response criteria were assessed every 2 months by computed tomography scanning and defined according to Response Evaluation Criteria in Solid Tumors (RECIST) 1.1.

### Exome sequencing

Whole-exome sequencing was performed on an Illumina HiSeq. 2000 system (San Diego, CA, USA) using the SureSelect Human All Exon v4 (51 Mb) capture protocol (Agilent Technologies, Santa Clara, CA, USA). Paired-end sequencing with a read length of 100 base pairs and 100X average on-target coverage was conducted. Sequencing reads were mapped to the human genome (hg19) using the Burrows-Wheeler Aligner MEM algorithm. Variants were called using SAMtools^24^, Genome Analysis Toolkit (GATK-lite version)^46^, and FreeBayes^47^. Variants were filtered with a genotype Q score of ≥30, ≥3 variant reads, variant allele fraction of ≥0.05, and read coverage of ≥10 reads. Somatic mutations were called at positions with matched normal coverage of ≥10 reads, normal variant allele fraction of ≤0.05, normal variant allele fraction <2X of the tumour variant fraction, and no more than two pooled normal samples (pooling of all matched normal samples) with a variant allele fraction of ≥0.20.

### CN analysis

DNA was prepared and loaded according to the manufacturer’s protocol on Affymetrix’s Genome-Wide Human SNP Array 6.0 (SNP6; Santa Clara, CA, USA). CEL files were used for CN analysis using three methods: (i) PICNIC^47^, for ploidy estimation; (ii) ASCAT^48^, for genome-wide visualization; and (iii) the Copy Number Inference Pipeline (CNIP) in GenePattern, for gene CNs. Three samples (5225AR, 4612S, and 7522S) failed to be completed using the PICNIC method. Candidate cancer genes included in the Sanger Cancer Census Gene list (http://cancer.sanger.ac.uk/census) with CN calls of ≥4 or <0.5 copies based on CNIP analysis were further confirmed using exome CN calls from CNVkit^49^. CN calls without matching support in SNP6 and exome data were not considered reliable.

### RNA sequencing analysis

RNA sequencing of all samples was conducted in four data delivery batches. RNA-Seq was performed on an Illumina HiSeq. 2000 with the Illumina TruSeq RNA Sample Preparation Kit v2. Paired-end sequencing with a read length of 100 bp and targeted read depth of 50 million reads/sample was performed. Sequencing reads were mapped to the human reference genome (B37.p5 including all alternative contigs) using GSNAP (Wu *et al*., version 2013-11-27) with NCBI annotation as of December 2013. Read counts were generated against exons annotated in NCBI gene models and then summarized at the gene level to provide a single number/gene/sample using a custom perl script. Data were filtered to remove genes with fewer than 10 counts across 80% of the samples from the analysis. The resulting data were quantile-normalised and summarised across samples. Clustering analysis was carried out using all genes (21,344 genes) and genes with relatively high signals (10,346 genes, signals >10 in all samples). All samples were grouped into three batches based on clustering analysis results.

Data analysis was carried out on log2-transformed signals using a proc mixed procedure in SAS (SAS Institute, Cary, NC, USA). The statistical model included sensitivity as a fixed effect and batch as a random effect. Samples from the same subject were treated by repeated measurement using the covariance matrix option toep. A small number of genes used the ar(1) option if the model could not converge with toep. The results of comparisons of interest were then derived from the statistical modelling outputs. Fold changes and mean signals were converted back to count levels.

### Fusion gene detection

BAM files generated by GSNAP (see above) were investigated for reads that had split alignments, identifying more than one gene either through normal splicing (indicated by an ‘N’ CIGAR) or through chimeric alignment (indicated by the ‘XT’ tag). Only fusions joining CDS in-frame and including at least 10 amino acids from both proteins were retained. Normal splicing reads for genes within 5 KB were discarded. Putative fusions previously found to occur in >1% of a large internal collection of non-cancer samples or in >10% of cancer samples were discarded as likely false-positives. Paired-end reads identifying putative gene fusions were also recorded. Potential fusions with ≥5 total reads (split and paired) were subjected to further analysis.

### Fusion gene confirmation

RNA from tumour and normal tissues were used to produce cDNA using the SuperScript VILO MasterMix (Invitrogen, Carlsbad, CA, USA; #11755-050). A forward primer upstream of the fusion junction (previously identified from RNA-Seq data) and reverse primer downstream of the fusion junction were designed using the online program Primer3 for PCR and Sanger sequencing primers. The primer sequences are NCOA4_F1: GAGCCTGAGAAGCATAAAGATTCC, RET_R1: CCCATACAATTTGATGACATGTGG, LMNA_F2: CAAAGTGCGTGAGGAGTTTAAGG, NTRK1_R2, and CACTGAAGTATTGTGGGTTCTCG. PCR was carried out using Advantage®2 Polymerase Mix (Clontech, Mountain View, CA, USA; #639201) with Advantage 2 PCR buffer and cycled at 95 °C for 2 min; 35 cycles of 95 °C for 30 s; 65 °C for 30 s, 72 °C for 30 s; and a final extension at 72 °C for 10 min. PCR was also performed for the *GAPDH* gene as a control (GAPDH_F: ATCCCATCACCATCTTCCAG, GAPDH_R: CCATCACGCCACAGTTTCC). PCR products were purified with a Wizard® SV Gel and PCR Clean-Up System (Promega, Madison, WI, USA; #A9281). Sequencing PCR was performed using an ABI BigDye Terminator v3.1 cycle sequencing kit (Life Technologies, Carlsbad, CA, USA; #4337457). The resulting products were run on an ABI 3730xl DNA analyser. All sequences were visually analysed with Sequencher (Gene Codes Corp., Ann Arbor, MI, USA).

PCR and Sanger sequencing primers used to confirm the identity the PDC line were as follows: SLC17A1_F: TACCACTCAGCCAGTCAAATACC, SLC17A1_R: TCTGTGGTGACACTAGAAAGTTGC; GTF2H5_F: TTGTTAACACTTGAGGCAGAGAGG, GTF2H5_R: CCAAATTACAGCCAACTGTTAAAGC.

### Gene set enrichment analysis

We performed gene set enrichment analysis using previously described signatures and well-characterized pathways in CRC^50^. These gene sets included canonical pathways, immune signatures, an immune and stromal cell admixture in tumour samples, and metabolic pathways. The signature score as an activation index was calculated using a combined Z score method^51,52^, which combines Z scores for each gene in a signature set for each sample. The Z score represents both the magnitude and relative direction of a signature’s expression. ESTIMATE R package was used to quantify immune and stromal cell infiltration^26^.

### Patient-derived cell culture

Fresh tissue specimens were extensively washed with serum-free RPMI 1640, minced, and enzymatically dissociated for 2 h at 37 °C with agitation in serum-free RPMI 1640 containing 0.4 mg/mL collagenase (Gibco, Grand Island, NY, USA), 0.5 mg/mL dispase (Gibco), and 0.2 mg/mL DNase I (Roche, Basel, Switzerland), as described previously^53^. The cells were cultured in RPMI 1640 supplemented with 10% foetal bovine serum. Experiments were conducted within four passages after PDC derivation.

### Cell proliferation assay

Cells were seeded at 5,000 cells per well in 100 μL of media in a 96-well plate, grown overnight, and then treated with different concentrations of drugs for 5 days prior to analysis using CellTiter-Glo (Promega; #G7572) according to the manufacturer’s instructions. All treatments were performed in duplicate, and four independent experiments were performed.

### Immunoblot analysis

Cells were treated with 75 µg/mL cetuximab and 1 M erlotinib, GDC-0994, BEZ-235, AZD-8055, and BKM-120 for 24 h and then lysed with RIPA lysis buffer + Halt protease/phosphatase inhibitor cocktail. Protein concentration was measured using the BCA protein assay. Equal amounts of protein were separated on a 4–12% Bis-Tris Criterion polyacrylamide gel (Bio-Rad, Hercules, CA, USA) in MOPS buffer under reducing conditions and then transferred to a nitrocellulose membrane. The membranes were blocked with Odyssey blocking buffer in Tris-buffered saline and probed overnight at 4 °C with the indicated primary antibodies diluted in Odyssey blocking buffer + 0.2% Tween-20. The phosphor- and total EGFR, AKT, S6RP, 4E-BP1, and ERK antibodies were obtained from Cell Signaling Technology (Danvers, MA, USA), while the β-actin antibody was from Sigma (St. Louis, MO, USA). IRDye secondary antibodies were from Li-Cor, and a Li-Cor Odyssey scanner was used for visualization (Lincoln, NE, USA).

### Real-time quantitative reverse transcription PCR

Real-time quantitative reverse transcription PCR (RT-qPCR) assays were performed using Taqman® probes for E-cadherin (CDH1; assay ID Hs01023895_m1), N-cadherin (CDH2; Hs00983056_m1), vimentin (Hs00958111_m1), and AXL (Hs01064444_m1). All reactions were performed in triplicate in 384-well plates following the manufacturer’s instructions and measured with a Quant Studio7 Flex (Thermo Fisher Scientific, Waltham, MA, USA). RT-qPCR analysis was performed using the comparative Ct (cycle threshold) method (2^−ΔΔ^Ct) using RNA18S5 as the control gene; the results are reported as the fold difference from matching normal tissue.

## Supplementary information

Supplementary Figure

Supplementary Tables

## Data Availability

The datasets generated during and/or analysed during the current study are available in the European Nucleotide Archive (ENA) repository, (Accession Number: PRJEB34338).
