# Supplementary material for: Genomic characterization of intrinsic and acquired resistance to cetuximab in colorectal cancer patients
Source: Sci Rep. 2019 Oct 25;9:15365. doi: 10.1038/s41598-019-51981-5 (PMC6814827; doi:10.1038/s41598-019-51981-5)
Supplement: Supplementary file 1 — Supplementary Figure [file 41598_2019_51981_MOESM1_ESM.pdf]

Supplementary Figures S1 – S5

Genomic characterization of intrinsic and acquired resistance to cetuximab in colorectal cancer patients

Steven M Bray[1#], Jeeyun Lee[2#], Seung Tae Kim[2#], Joon Young Hur[2], Philip J Ebert[1], John N Calley[1], Isabella H Wulur[1], Thejaswini Gopalappa[1], Swee Seong Wong[1], Hui-Rong Qian[1], Jason C Ting[1], Jiangang Liu[1], Melinda D Willard[1], Ruslan D Novosiadly[1], Young Suk Park[2], Joon Oh Park[2], Ho Yeong Lim[2], Won Ki Kang[2], Amit Aggarwal[1], Hee-Cheol Kim[3*] and Christoph Reinhard[1*]

1 Eli Lilly and Company, Lilly Research Laboratories, Oncology Discovery Research, Indianapolis, IN, USA
2 Division of Hematology-Oncology, Samsung Medical Center, Sungkyunkwan University School of Medicine, Seoul, Korea
3 Department of Surgery, Samsung Medical Center, Sungkyunkwan University School of Medicine, Seoul, Korea

[#]Co-first authors
*Co-corresponding authors

Figure S1.

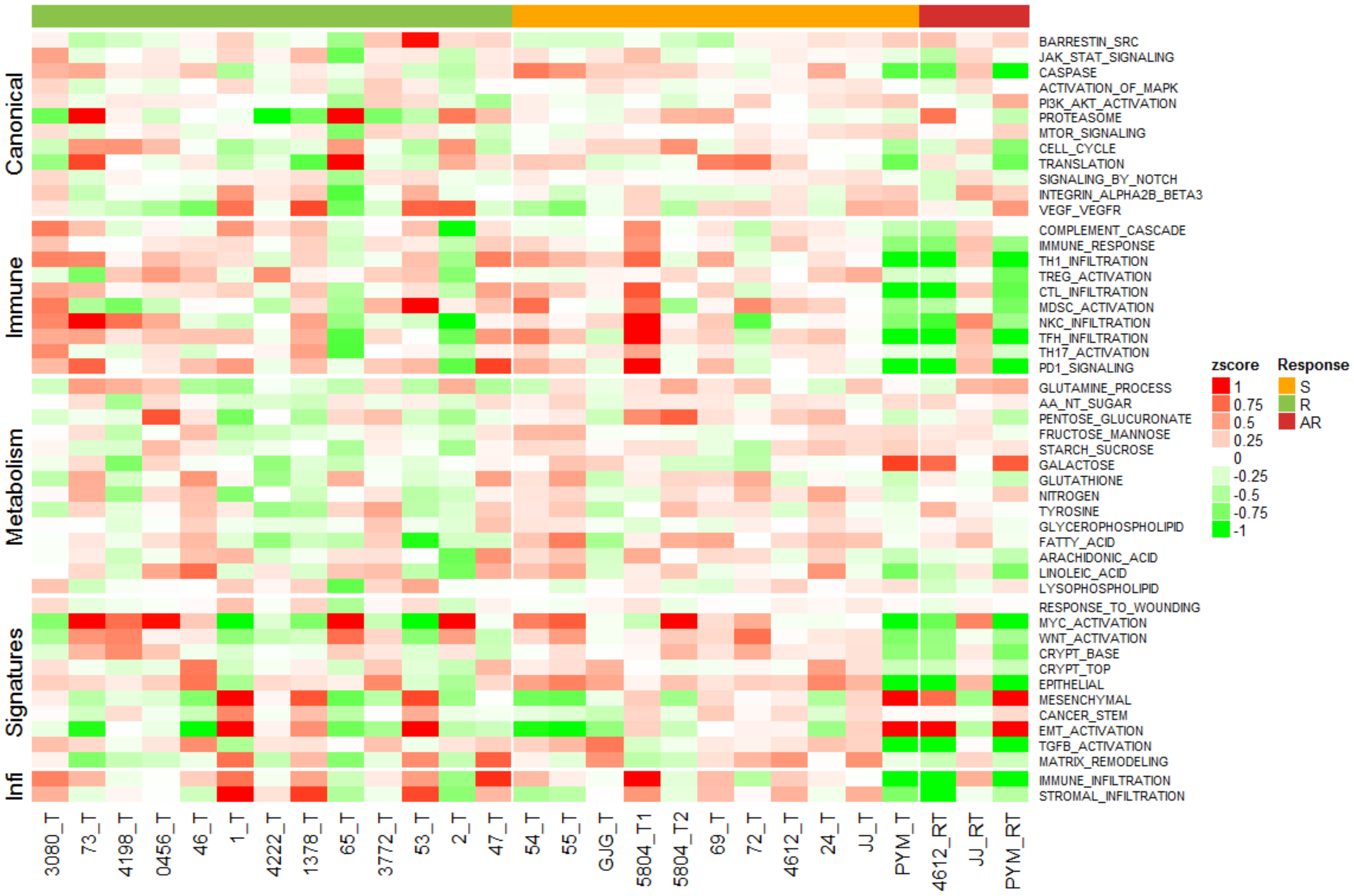

Figure S1. Heatmap showing enrichment of gene set expression signatures for a panel of previously published molecular signatures without hierarchical clustering (see Methods). The Z score represents both the magnitude and relative direction of a signature's expression. Three pairs of sensitive and acquired resistant tumors are indicated by matched blue arrows. Baseline tumor (T), Acquired Resistant Tumor (RT).

Figure S2.

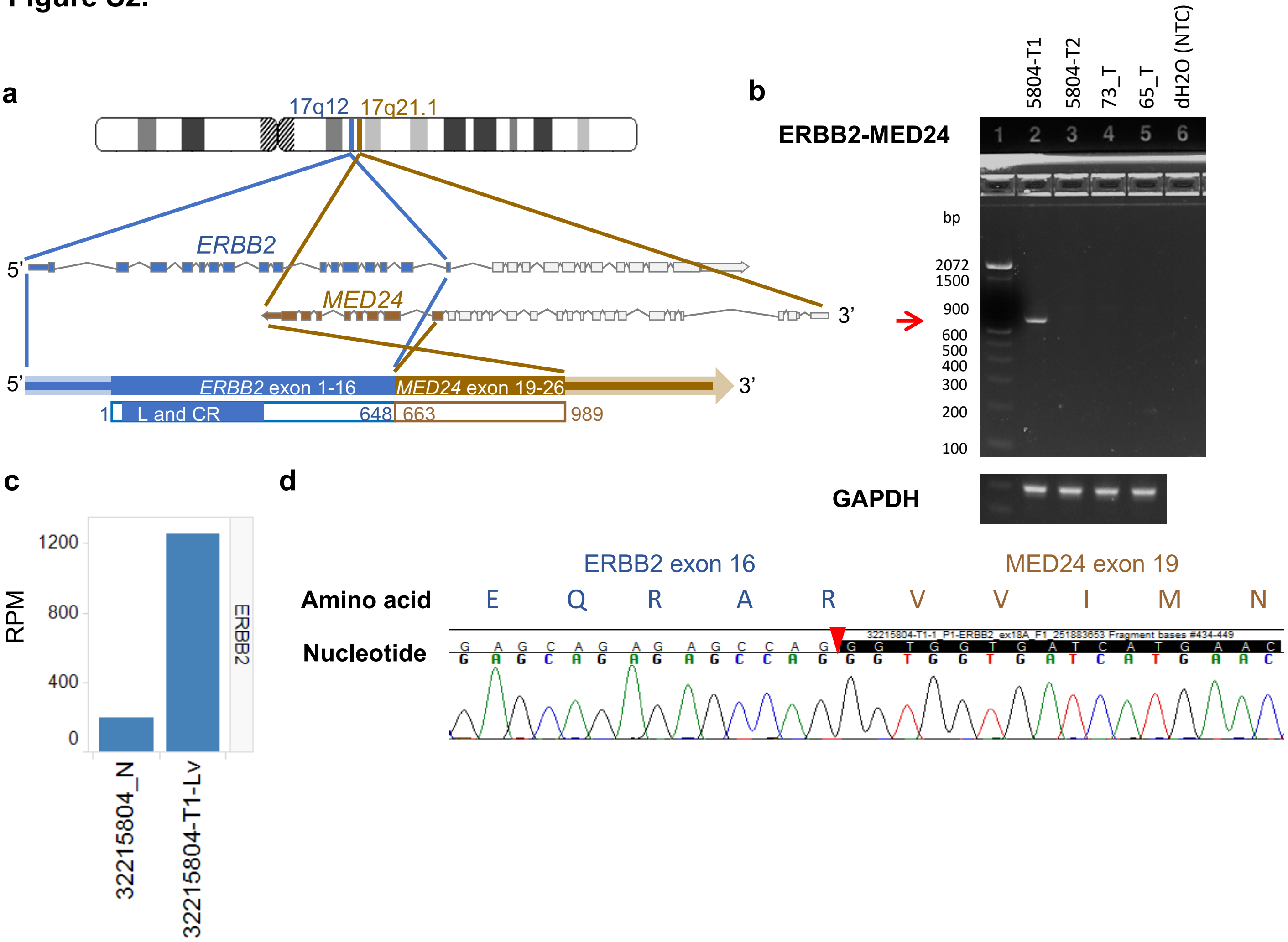

Figure S2. Characterization of ERBB2-MED24 Gene Fusion
(a) Schematic depiction of the gene fusion detected by RNA-Seq, indicating the genomic position, resulting fusion mRNA, and predicted protein consequences (with key protein domains indicated). **(b)** RT-PCR of RNA from tumor or adjacent normal tissue with primers upstream and downstream of the gene fusion breakpoint. NTC – no template control. Red arrow indicates the size of the expected PCR product. **(c)** Overexpression of the ERBB2 gene partner in the tumor relative to the adjacent normal tissue. ERBB2 overexpression is likely due to gene amplification and not the gene fusion event (data not shown). RPM – Reads Per Million. **(d)** Sanger sequencing of the RT-PCR product from **b.** Red arrowhead shows the position of the fusion junction and in-frame amino acid sequence.

Figure S3.

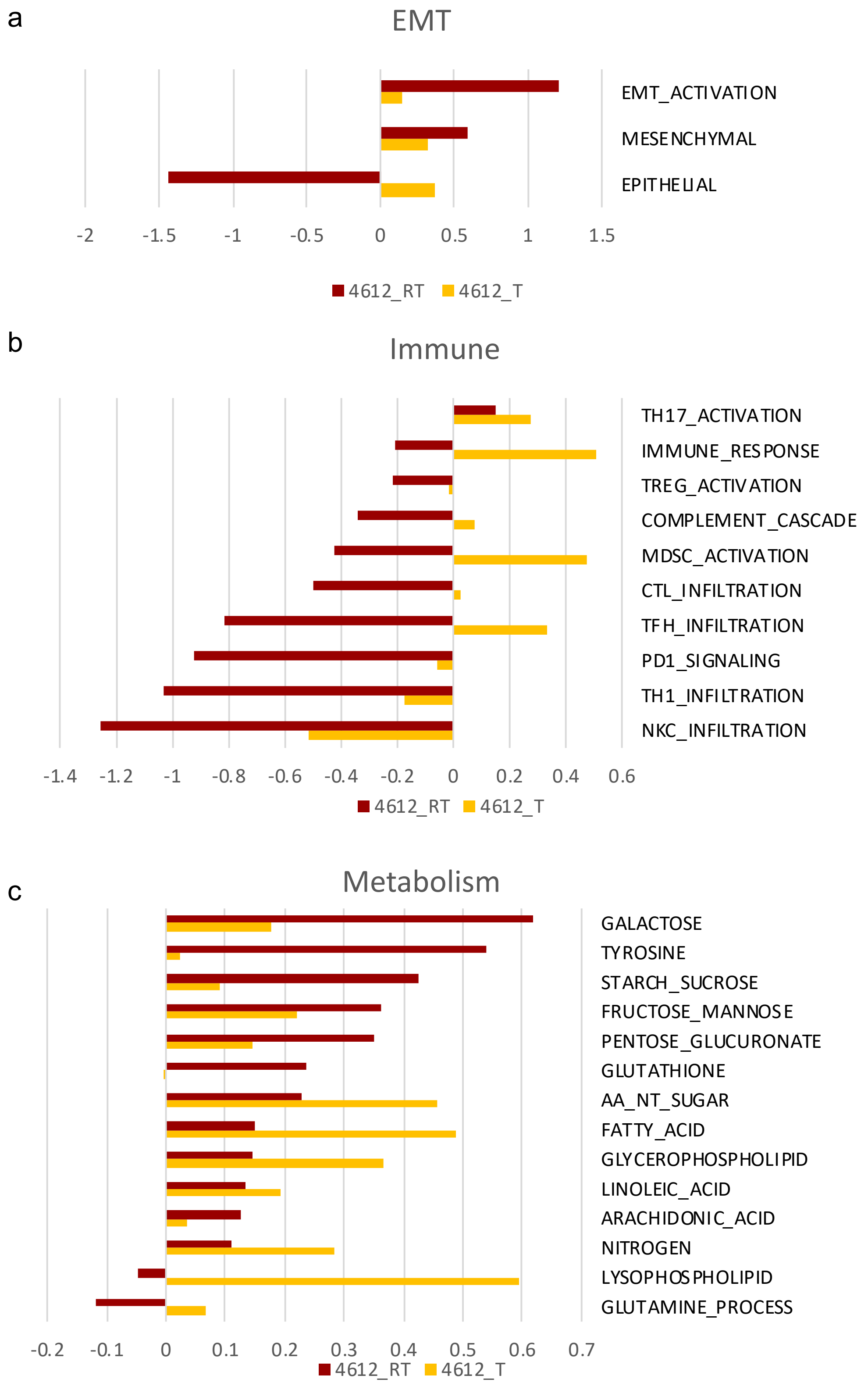

Figure S3. The bar charts show the summarized Z score between 4612_T and 4612_RT for EMT, Immune, and Metabolism molecular signatures. Baseline tumor (T) and acquired-resistant tumor (RT). Signatures are from Guinney et al, 2015.

Figure S4.

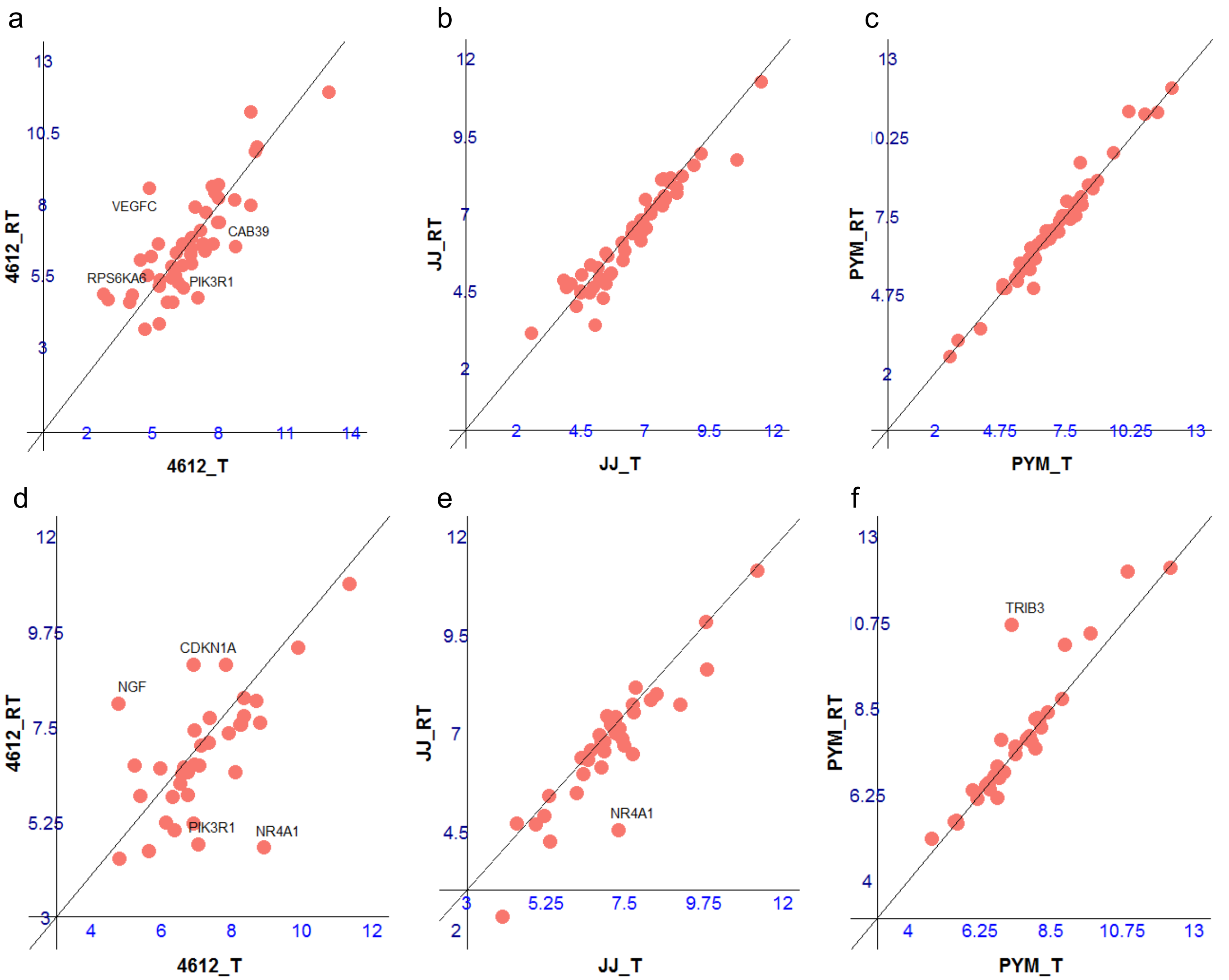

Figure S4. The scatter plots show the expression differences between 4612_T and 4612_RT, JJ_T and JJ_RT, and PYM_T and PYM_RT for KEGG “MTOR_SIGNALING” **(a-c)** and REACTOME “PI3K_AKT_ACTIVATION” pathway genes **(d-f)** (Guinney et al, 2015). A diagonal line is plotted for comparison. The pathway genes showing > 4-fold change difference of expression between primary tumor and acquired resistance tumor are labeled. Baseline tumor (T), Acquired Resistant Tumor (RT).

Figure S5.

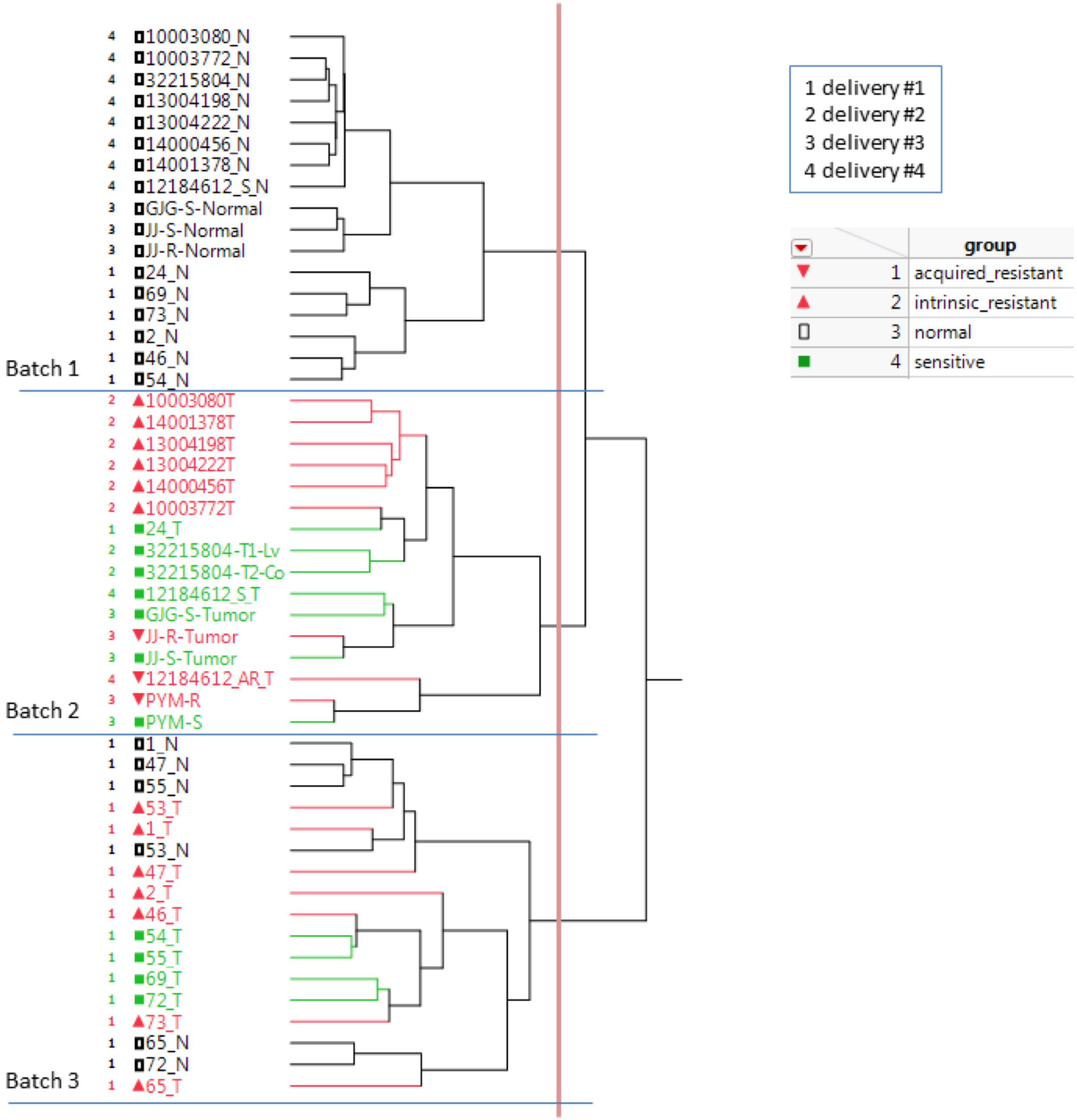

Figure S5. RNA-Seq Clustering into Batches by Data Delivery and Tissue Type
All RNA-Seq samples were grouped into 3 batches based on clustering analysis results and data generation/delivery dates. Batch 1 shows clustering of only adjacent normal tissues from multiple data generation/delivery dates. Batch 2 contains cetuximab sensitive and resistant tumors also from multiple data delivery dates. Batch 3 is a mix of normal and tumor samples all clustering from the first data generation/delivery date. Final statistical model included sensitivity as a fixed effect and calculated batch as a random effect.
